# Impact of the DNA Damage Response on Human Papillomavirus Chromatin

**DOI:** 10.1371/journal.ppat.1005613

**Published:** 2016-06-16

**Authors:** Dipendra Gautam, Cary A. Moody

**Affiliations:** Lineberger Comprehensive Cancer Center and Department of Microbiology and Immunology, University of North Carolina at Chapel Hill, Chapel Hill, North Carolina, United States of America; University of Kentucky, UNITED STATES

## Introduction

The fidelity of replication is regulated by the DNA damage response (DDR), an elaborate signaling network of proteins that detect, signal, and repair DNA lesions. While some viruses have evolved mechanisms to avoid or eliminate DNA repair machinery, others exploit the DDR to replicate their genomes [1]. Recent studies indicate that the DDR facilitates productive replication of human papillomaviruses (HPV) [2–8]. The ability of cells to detect and repair DNA breaks is dependent on the reorganization of surrounding chromatin [9]. The importance of histone post-translational modifications and chromatin remodeling proteins in recruitment of repair factors to DNA breaks is becoming increasingly clear. HPV genomes are histone-associated in the virion and exhibit a nucleosome pattern similar to that of cellular DNA in infected cells [10,11]. HPV chromatin is subject to histone modifications, likely important in ensuring the correct temporal expression of viral genes through the life cycle [12,13]. However, the assembly of DNA repair factors in large complexes at HPV replication centers raises the intriguing possibility that viral chromatin may also be subject to the changing chromatin dynamics associated with the DDR, facilitating efficient productive replication through DNA repair mechanisms.

## The Life Cycle of HPV

HPVs are small, double-stranded DNA viruses that exhibit a strict tropism for the mucosal or cutaneous stratified squamous epithelium. Mucosal HPV types are grouped into high-risk and low-risk categories based on their association with cancer. Outcomes of HPV infection can range from asymptomatic to a wide range of benign papillomas or warts. However, high-risk HPV types are the etiological agent of cervical cancer and other anogenital malignancies as well as an increasing number of oropharyngeal cancers [14].

The HPV life cycle has evolved to contend with different cell states found in a differentiating epithelium and relies on cellular factors [15]. HPV infects basal cells of the stratified epithelium, in which viral genomes are maintained as episomes at low copy number, with low levels of gene expression. In contrast, epithelial differentiation triggers the productive phase of the life cycle, resulting in viral genome amplification to thousands of copies per cell, late gene expression, and virion assembly. Paradoxically, HPV must amplify its genomes in differentiated cells that have exited the cell cycle. The viral E6 and E7 proteins circumvent this problem by targeting cell cycle checkpoint proteins (e.g., p53 and Rb, respectively) for degradation, pushing cells back into the cell cycle. Viral genome amplification is thought to follow cellular DNA synthesis as cells transition from S phase to a G2-like phase [16], providing cellular factors necessary for viral replication. While maintenance replication occurs via a bi-directional theta mode, increasing evidence suggests that productive viral replication occurs in a manner distinct from that found in undifferentiated cells [17]. Multiple studies support the idea that HPV activates an ataxia-telangiectasia mutated (ATM)-dependent DDR to amplify viral genomes in a recombination-dependent manner, which is supported through the recruitment of DDR repair proteins to viral replication compartments [2,3,7,18].

## HPV Activates the DNA Damage Response to Facilitate Viral Replication through Homologous Recombination

ATM is a serine/threonine kinase belonging to the PIKK family, which also includes DNA-PK (DNA-dependent protein kinase) and ATR (ATM and Rad3-related) [19]. ATM and DNA-PK are activated primarily in response to double-strand breaks (DSBs), while ATR responds to single-stranded DNA (ssDNA) that occurs upon resection of DSBs, or results from stalled replication forks. Once activated, these kinases initiate a signal transduction cascade resulting in activation of cell cycle checkpoints and recruitment of DNA repair factors to damaged DNA [20]. A seminal study in the HPV field demonstrated that ATM activation is required for productive replication of high-risk HPV31, but not for episomal maintenance [2]. Subsequent studies demonstrated that components of the ATM response are recruited to HPV replication sites (H2AX, Chk2, RPA, MRN complex [Mre11, Rad50, Nbs1], 53BP1, BRCA1, Rad51) [4,5,21–23], suggesting that HPV utilizes ATM activity to drive productive replication through DSB repair mechanisms. Both E7 and the viral helicase E1 can independently activate the ATM response and may have distinct roles in maintaining ATM activity in HPV-infected cells during various stages of the viral life cycle [2,21,22,24]. Several studies have shown that the ATR pathway is also active in HR-HPV-positive cells and can be activated in an E7- or E1-dependent manner [2,8,21,24]. ATR and its effector kinase Chk1 are required to stabilize replication forks in response to replication stress. Multiple factors from the ATR pathway localize to HPV replication compartments [21,24,25], and recent studies demonstrated that inhibition of ATR and Chk1 blocks productive replication [8]. Overall, these studies suggest that HPV manipulates both the ATM and ATR arms of the DDR in order to promote viral genome stability and ensure the efficient amplification of viral genomes through DNA repair mechanisms.

Eukaryotic cells repair DSBs by non-homologous end joining (NHEJ) or homologous recombination (HR) [26]. NHEJ is a low-fidelity repair process carried out by DNA-PK that occurs predominantly in G1 phase. The HR pathway requires ATM activity, provides accurate repair of DSBs by using a sister chromatid as a template, and is restricted to S and G2 phases. HR requires the resection of DSBs, which is initiated by ATM-dependent phosphorylation of the CtIP endonuclease as well as BRCA1-mediated inhibition of the resection inhibitor 53BP1 and the recruitment of CtIP to the MRN resection complex. Additional resection yields 3′-ssDNA overhangs that are coated by the ssDNA binding complex RPA, which is replaced by the recombinase Rad51. The Rad51 nucleofilament mediates homology search in the sister chromatid, followed by strand invasion into the homologous template.

The requirement of ATM activity for productive HPV replication, as well as the localization of HR repair factors (ATM, MRN complex, RPA, Rad51, BRCA1) to viral replication compartments, suggests that replication occurs in a recombination-dependent manner [18]. Indeed, studies have shown that the MRN complex, BRCA1, and Rad51 are required for productive replication [5,6]. Inhibition of Mre11’s endonuclease activity blocks viral genome amplification [5], indicating that resection, which is required for Rad51 loading, is necessary for viral replication. In support of this, Rad51 binding to viral genomes increases during productive replication, and inhibition of Rad51’s DNA binding activity prevents viral DNA synthesis [6]. In contrast to the recruitment of HR repair factors, the classic NHEJ factor DNA-PK does not localize to HPV genomes [4], suggesting that NHEJ does not make a significant contribution to HPV replication, though this has not been specifically examined. Studies involving SV40 have demonstrated that ATM activity is important for the recruitment of HR factors to viral DNA and the inhibition of NHEJ-mediated repair of viral replication products [27]. The studies described above suggest a similar scenario for HPV, with ATM activity directing repair to HR rather than NHEJ on viral genomes. In addition, these studies raise the question of whether repair factor recruitment to viral DNA follows the same hierarchy of signaling/recruitment events associated with the cellular DDR, which requires a dynamic chromatin response.

## Chromatin Modifications Facilitate Access of Repair Factors to DNA Lesions

**Fig 1:**
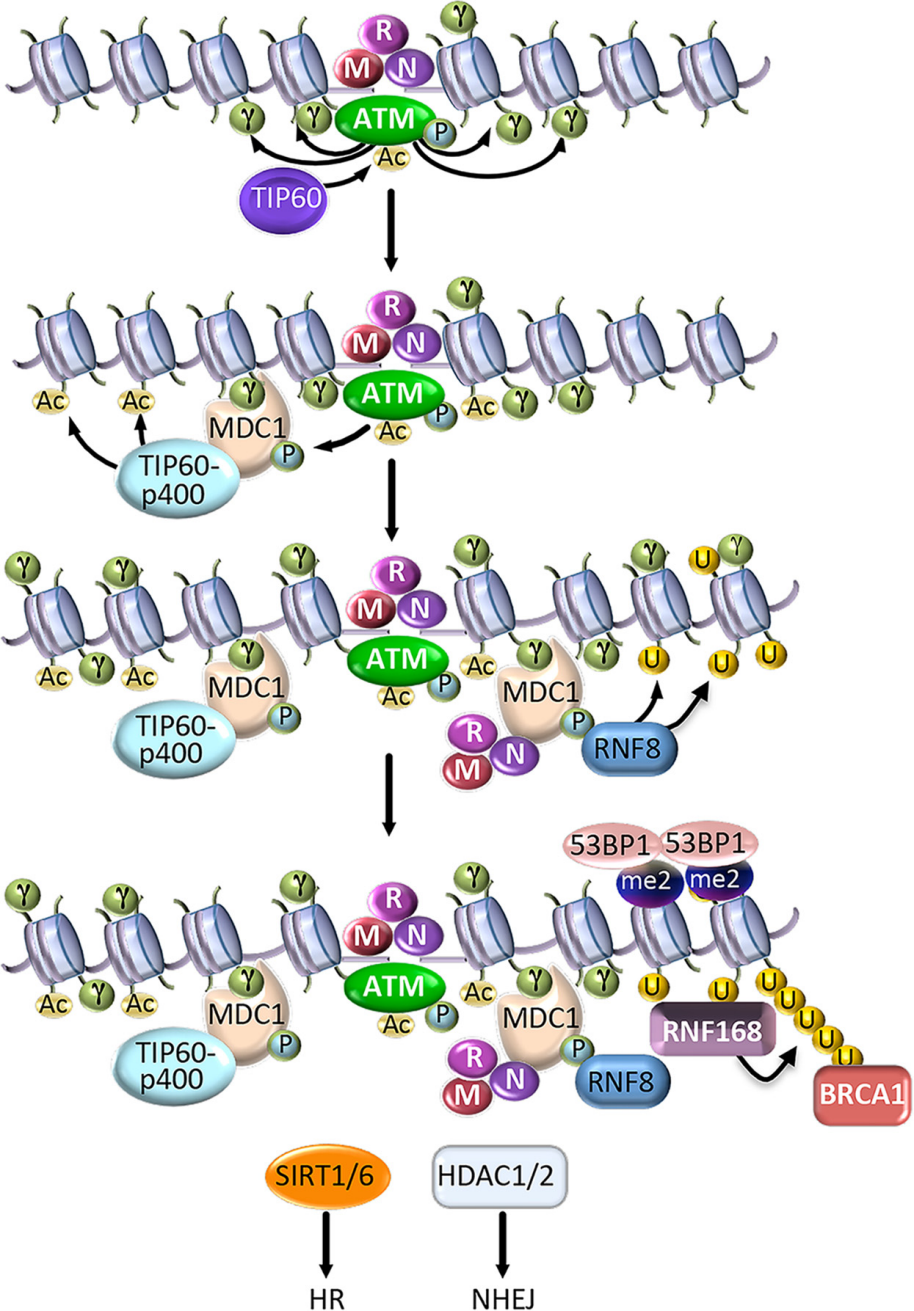
Chromatin dynamics in response to double strand break (DSB) formation. The MRN complex rapidly senses DNA breaks and, together with TIP60 acetyltransferase, recruits and activates the ATM kinase through auto-phosphorylation on Ser1981 (depicted as P) and acetylation (depicted as Ac), respectively. ATM initiates a signaling cascade by phosphorylation of histone H2AX on Ser139, forming γH2AX at the DNA lesion (depicted as γ). γH2AX serves as a docking site for recruitment of the scaffolding protein MDC1. MDC1 is phosphorylated by ATM and recruits multiple DDR factors. MDC1 recruits the MRN complex through binding of Nbs1, allowing further recruitment of ATM and the spread of γH2AX away from the DSB site. MDC1 also recruits the Nu4A complex, consisting of the p400 SWI/SNF ATPase and TIP60, which allows for acetylation of histone H4K16. Phospho-MDC1 serves as a docking site for the ubiquitin ligase RNF8, which ubiquitylates H2A/H2AX (depicted as U). Ubiquitylation triggers recruitment of the ubiquitin ligase RNF168, which binds and amplifies the ubiquitin conjugates initiated by RNF8, resulting in the loading of BRCA1 and 53BP1, which participate in DSB repair. 53BP1 is a bivalent histone code reader whose stable retention at DSBs requires the recognition of the DNA damage inducible mark H2AK15ub as well as nucleosomes modified with H3K20me2 (depicted as me2) [30]. The recruitment of SIRT1 and SIRT6 stimulates HR factor recruitment, while recruitment of HDAC1 and HDAC2 promotes recruitment of NHEJ factors.

DNA damage induces structural changes in chromatin that are orchestrated through ATP-dependent remodeling complexes as well as post-translational modifications of histones and histone-binding proteins (i.e., phosphorylation, acetylation, ubiquitylation) [9,20]. The general chromatin response to DSB formation is outlined in Fig 1 and discussed briefly below. In response to DSBs, ATM is activated via recruitment to DNA lesions by the MRN complex and acetylation by TIP60 [28]. At DSBs, ATM rapidly phosphorylates the histone variant H2AX on S139, forming γH2AX [29]. γH2AX initiates the assembly of repair factors at DNA lesions in a highly regulated manner, with one key function being the recruitment of the scaffolding protein MDC1. MDC1 recruits the MRN complex, further amplifying the DDR response. MDC1 also promotes recruitment of the E3 ubiquitin ligases Ring Finger 8 (RNF8) and RNF168. Together, RNF8/RNF168 catalyze non-proteolytic K63-linked ubiquitin chains on H2A/H2AX, facilitating the binding of BRCA1 as well as 53BP1 [30]. The interplay between 53BP1 and BRCA1 fine-tunes the DSB repair pathway utilized, with BRCA1 promoting HR through initiating end resection in S/G2 and 53BP1 committing repair to NHEJ by blocking BRCA1 accumulation and end resection in G1 [26].

Acetylation of histones in the vicinity of DSBs also regulates the recruitment of repair factors to DNA lesions. MDC1 recruits NuA4, a multi-subunit remodeling complex containing TIP60 as well as the p400 SWI/SNF ATPase [31]. p400 decreases nucleosome stability at DSBs, allowing for acetylation of histone H4 by TIP60. p400/TIP60 catalyze a shift from repressive to open, acetylated chromatin. Inactivation of either TIP60 or p400 blocks histone ubiquitylation by RNF8/RNF168, inhibiting loading of BRCA1, 53BP1, and Rad51 onto chromatin. Multiple deacetylases also localize to DSB sites, including SIRT1, SIRT6, HDAC1, and HDAC2. SIRT6 and SIRT1 have been reported to promote recruitment of HR repair factors to DSBs [32,33]. In contrast, HDAC1 and HDAC2 prevent the accumulation of BRCA1 at DSBs and promote the retention of 53BP1 through targeting H4 acetylation, directing repair to NHEJ over HR [34,35].

## Is HPV Chromatin Subject to DDR-Associated Modifications?

Several recent studies support the idea that HPV chromatin is modified by the DDR. Gillespie et al. demonstrated that γH2AX localizes to HPV replication compartments, with γH2AX foci size increasing with productive replication [4]. Importantly, γH2AX was found to bind viral DNA, suggesting that γH2AX may serve to assemble repair factors at viral replication sites. In support of this, DDR components that rely on γH2AX for recruitment to DNA breaks, including 53BP1, Nbs1, BRCA1, and Rad51, also localize to HPV replication compartments [4,22,23,36]. Given that the recruitment of 53BP1 as well as BRCA1 to DSBs can occur in an ubiquitin-dependent manner, these results also suggest that RNF8/RNF168 may localize to viral DNA. However, the impact of HPV infection on RNF8/RNF168 expression, localization, and function has not been determined.

DDR-associated acetyltransferases and deactylases have also been linked to efficient HPV replication. Hong et al. recently demonstrated that TIP60 is active in HPV-positive cells and is required for productive viral replication, presumably through facilitating ATM activation [3]. TIP60 can also influence the repair pathway of choice to HR through H4 acetylation and attenuation of 53BP1 binding [34], and TIP60 could potentially exert a similar effect on HPV chromatin. Recent studies also support a role for the SIRT1 deactylase in the recruitment of HR factors to HPV genomes. In response to DNA damage, SIRT1 binds in the vicinity of DSBs and recruits Nbs1 and Rad51 in an ATM- and γH2AX-dependent manner [32]. SIRT1 is up-regulated in HPV-positive cells and is recruited to multiple sites in the viral genome [36,37]. Importantly, in the absence of SIRT1, Nbs1 and Rad51 no longer bind to viral DNA, and productive viral replication is blocked [36]. SIRT1, as well as TIP60, may modify viral chromatin, ensuring the recruitment of HR repair factors that facilitate productive viral replication. The ability of high-risk HPV E7 proteins to bind type 1 HDACs (HDACs 1–3) has also been reported to directly impact viral replication, with mutation of the E7 HDAC binding domain preventing episomal maintenance and blocking productive replication [38,39]. While the effect of the E7/HDAC interaction on viral chromatin is currently unknown, it is possible that E7 sequesters HDACs from viral genomes, in turn preventing chromatin modifications that would drive the recruitment of NHEJ factors, and instead promotes HR repair factor localization to viral replication compartments. Further understanding of the impact of DDR-associated acetyltransferases and deacetylases on HPV chromatin and the recruitment of repair factors to viral replication sites will be an important area of future investigation.

## Conclusions

HPV requires ATM activity and the recruitment of HR factors to viral DNA for productive replication. The binding of γH2AX to viral DNA suggests that HPV-induced activation of ATM results in chromatin changes that promote the recruitment of HR rather than NHEJ factors to viral replication centers. Understanding how viral chromatin modifications are altered by the DDR and whether this deviates from the normal response to DNA damage will provide further insight into the mechanisms by which viral replication is controlled. Activation of ATM, phosphorylation of H2AX, and the recruitment of DNA repair factors to viral replication centers are observed upon infection with multiple DNA viruses, including SV40, HCMV, HSV-1, KSHV, EBV, MCPyV, and γHV68 [40,41]. Determining if DDR-associated changes to viral chromatin serve as a common means to facilitate the recruitment of repair factors to viral DNA and promote viral replication provides an exciting avenue of future investigation.

## References

[1] Weitzman MD, Weitzman JB. What's the damage? The impact of pathogens on pathways that maintain host genome integrity. Cell Host Microbe. 2014;15(3):283–94. 10.1016/j.chom.2014.02.010 24629335 PMC4501477

[2] Moody CA, Laimins LA. Human papillomaviruses activate the ATM DNA damage pathway for viral genome amplification upon differentiation. PLoS Pathog. 2009;5(10):e1000605 10.1371/journal.ppat.1000605 19798429 PMC2745661

[3] Hong S, Dutta A, Laimins LA. The acetyltransferase Tip60 is a critical regulator of the differentiation-dependent amplification of human papillomaviruses. J Virol. 2015;89(8):4668–75. 10.1128/JVI.03455-14 25673709 PMC4442364

[4] Gillespie KA, Mehta KP, Laimins LA, Moody CA. Human papillomaviruses recruit cellular DNA repair and homologous recombination factors to viral replication centers. J Virol. 2012;86(17):9520–6. 10.1128/JVI.00247-12 22740399 PMC3416172

[5] Anacker DC, Gautam D, Gillespie KA, Chappell WH, Moody CA. Productive replication of human papillomavirus 31 requires DNA repair factor Nbs1. J Virol. 2014;88(15):8528–44. 10.1128/JVI.00517-14 24850735 PMC4135936

[6] Chappell WH, Gautam D, Ok ST, Johnson BA, Anacker DC, Moody CA. Homologous Recombination Repair Factors Rad51 and BRCA1 Are Necessary for Productive Replication of Human Papillomavirus 31. J Virol. 2015;90(5):2639–52. 10.1128/JVI.02495-15 . 26699641 PMC4810724

[7] Hong S, Laimins LA. The JAK-STAT transcriptional regulator, STAT-5, activates the ATM DNA damage pathway to induce HPV 31 genome amplification upon epithelial differentiation. PLoS Pathog. 2013;9(4):e1003295 10.1371/journal.ppat.1003295 23593005 PMC3616964

[8] Hong S, Cheng S, Iovane A, Laimins LA. STAT-5 Regulates Transcription of the Topoisomerase IIbeta-Binding Protein 1 (TopBP1) Gene To Activate the ATR Pathway and Promote Human Papillomavirus Replication. MBio. 2015;6(6):e02006–15. 10.1128/mBio.02006-15 26695634 PMC4701836

[9] Papamichos-Chronakis M, Peterson CL. Chromatin and the genome integrity network. Nat Rev Genet. 2013;14(1):62–75. 10.1038/nrg3345 23247436 PMC3731064

[10] Favre M, Breitburd F, Croissant O, Orth G. Chromatin-like structures obtained after alkaline disruption of bovine and human papillomaviruses. J Virol. 1977;21(3):1205–9. 191643 10.1128/jvi.21.3.1205-1209.1977 PMC515661

[11] Stunkel W, Bernard HU. The chromatin structure of the long control region of human papillomavirus type 16 represses viral oncoprotein expression. J Virol. 1999;73(3):1918–30. 9971771 10.1128/jvi.73.3.1918-1930.1999 PMC104433

[12] del Mar Pena LM, Laimins LA. Differentiation-dependent chromatin rearrangement coincides with activation of human papillomavirus type 31 late gene expression. J Virol. 2001;75(20):10005–13. 10.1128/JVI.75.20.10005–10013.2001 11559836 PMC114575

[13] Wooldridge TR, Laimins LA. Regulation of human papillomavirus type 31 gene expression during the differentiation-dependent life cycle through histone modifications and transcription factor binding. Virology. 2008;374(2):371–80. 10.1016/j.virol.2007.12.011 18237759 PMC2410142

[14] zur Hausen H. Papillomaviruses in the causation of human cancers—a brief historical account. Virology. 2009;384(2):260–5. 10.1016/j.virol.2008.11.046 . 19135222

[15] Moody CA, Laimins LA. Human papillomavirus oncoproteins: pathways to transformation. Nat Rev Cancer. 2010;10(8):550–60. 10.1038/nrc2886 . 20592731

[16] Banerjee NS, Wang HK, Broker TR, Chow LT. Human papillomavirus (HPV) E7 induces prolonged G2 following S phase reentry in differentiated human keratinocytes. J Biol Chem. 2011;286(17):15473–82. 10.1074/jbc.M110.197574 21321122 PMC3083224

[17] Flores ER, Lambert PF. Evidence for a switch in the mode of human papillomavirus type 16 DNA replication during the viral life cycle. J Virol. 1997;71(10):7167–79. 9311789 10.1128/jvi.71.10.7167-7179.1997 PMC192056

[18] Sakakibara N, Chen D, McBride AA. Papillomaviruses use recombination-dependent replication to vegetatively amplify their genomes in differentiated cells. PLoS Pathog. 2013;9(7):e1003321 10.1371/journal.ppat.1003321 23853576 PMC3701714

[19] Ciccia A, Elledge SJ. The DNA damage response: making it safe to play with knives. Mol Cell. 2010;40(2):179–204. 10.1016/j.molcel.2010.09.019 20965415 PMC2988877

[20] Polo SE, Jackson SP. Dynamics of DNA damage response proteins at DNA breaks: a focus on protein modifications. Genes Dev. 2011;25(5):409–33. 10.1101/gad.2021311 21363960 PMC3049283

[21] Sakakibara N, Mitra R, McBride AA. The papillomavirus E1 helicase activates a cellular DNA damage response in viral replication foci. J Virol. 2011;85(17):8981–95. 10.1128/JVI.00541-11 21734054 PMC3165833

[22] Fradet-Turcotte A, Bergeron-Labrecque F, Moody CA, Lehoux M, Laimins LA, Archambault J. Nuclear accumulation of the papillomavirus E1 helicase blocks S-phase progression and triggers an ATM-dependent DNA damage response. J Virol. 2011;85(17):8996–9012. 10.1128/JVI.00542-11 21734051 PMC3165840

[23] Sakakibara N, Chen D, Jang MK, Kang DW, Luecke HF, Wu SY, et al Brd4 is displaced from HPV replication factories as they expand and amplify viral DNA. PLoS Pathog. 2013;9(11):e1003777 10.1371/journal.ppat.1003777 24278023 PMC3836737

[24] Reinson T, Toots M, Kadaja M, Pipitch R, Allik M, Ustav E, et al Engagement of the ATR-dependent DNA damage response at the human papillomavirus 18 replication centers during the initial amplification. J Virol. 2013;87(2):951–64. 10.1128/JVI.01943-12 23135710 PMC3554080

[25] Gauson EJ, Donaldson MM, Dornan ES, Wang X, Bristol M, Bodily JM, et al Evidence supporting a role for TopBP1 and Brd4 in the initiation but not continuation of human papillomavirus 16 E1/E2-mediated DNA replication. J Virol. 2015;89(9):4980–91. 10.1128/JVI.00335-15 25694599 PMC4403487

[26] Ceccaldi R, Rondinelli B, D'Andrea AD. Repair Pathway Choices and Consequences at the Double-Strand Break. Trends Cell Biol. 2016;26(1):52–64. 10.1016/j.tcb.2015.07.009 . 26437586 PMC4862604

[27] Sowd GA, Mody D, Eggold J, Cortez D, Friedman KL, Fanning E. SV40 utilizes ATM kinase activity to prevent non-homologous end joining of broken viral DNA replication products. PLoS Pathog. 2014;10(12):e1004536 10.1371/journal.ppat.1004536 25474690 PMC4256475

[28] Sun Y, Jiang X, Chen S, Fernandes N, Price BD. A role for the Tip60 histone acetyltransferase in the acetylation and activation of ATM. Proc Natl Acad Sci U S A. 2005;102(37):13182–7. 10.1073/pnas.0504211102 16141325 PMC1197271

[29] Bakkenist CJ, Kastan MB. Chromatin perturbations during the DNA damage response in higher eukaryotes. DNA Repair (Amst). 2015;36:8–12. 10.1016/j.dnarep.2015.09.002 26391293 PMC4727245

[30] Luijsterburg MS, van Attikum H. Close encounters of the RNF8th kind: when chromatin meets DNA repair. Curr Opin Cell Biol. 2012;24(3):439–47. 10.1016/j.ceb.2012.03.008 . 22464734

[31] Gursoy-Yuzugullu O, House N, Price BD. Patching Broken DNA: Nucleosome Dynamics and the Repair of DNA Breaks. J Mol Biol. 2015 10.1016/j.jmb.2015.11.021 . 26625977 PMC4860187

[32] Oberdoerffer P, Michan S, McVay M, Mostoslavsky R, Vann J, Park SK, et al SIRT1 redistribution on chromatin promotes genomic stability but alters gene expression during aging. Cell. 2008;135(5):907–18. 10.1016/j.cell.2008.10.025 19041753 PMC2853975

[33] Kaidi A, Weinert BT, Choudhary C, Jackson SP. Human SIRT6 promotes DNA end resection through CtIP deacetylation. Science. 2010;329(5997):1348–53. 10.1126/science.1192049 20829486 PMC3276839

[34] Tang J, Cho NW, Cui G, Manion EM, Shanbhag NM, Botuyan MV, et al Acetylation limits 53BP1 association with damaged chromatin to promote homologous recombination. Nat Struct Mol Biol. 2013;20(3):317–25. 10.1038/nsmb.2499 23377543 PMC3594358

[35] Miller KM, Tjeertes JV, Coates J, Legube G, Polo SE, Britton S, et al Human HDAC1 and HDAC2 function in the DNA-damage response to promote DNA nonhomologous end-joining. Nat Struct Mol Biol. 2010;17(9):1144–51. 10.1038/nsmb.1899 20802485 PMC3018776

[36] Langsfeld ES, Bodily JM, Laimins LA. The Deacetylase Sirtuin 1 Regulates Human Papillomavirus Replication by Modulating Histone Acetylation and Recruitment of DNA Damage Factors NBS1 and Rad51 to Viral Genomes. PLoS Pathog. 2015;11(9):e1005181 10.1371/journal.ppat.1005181 26405826 PMC4583417

[37] Allison SJ, Jiang M, Milner J. Oncogenic viral protein HPV E7 up-regulates the SIRT1 longevity protein in human cervical cancer cells. Aging (Albany NY). 2009;1(3):316–27. 20157519 10.18632/aging.100028 PMC2806013

[38] Longworth MS, Laimins LA. The binding of histone deacetylases and the integrity of zinc finger-like motifs of the E7 protein are essential for the life cycle of human papillomavirus type 31. J Virol. 2004;78(7):3533–41. 15016876 10.1128/JVI.78.7.3533-3541.2004 PMC371089

[39] Longworth MS, Wilson R, Laimins LA. HPV31 E7 facilitates replication by activating E2F2 transcription through its interaction with HDACs. EMBO J. 2005;24(10):1821–30. 10.1038/sj.emboj.7600651 15861133 PMC1142589

[40] Lilley CE, Chaurushiya MS, Weitzman MD. Chromatin at the intersection of viral infection and DNA damage. Biochim Biophys Acta. 2010;1799(3–4):319–27. 10.1016/j.bbagrm.2009.06.007 19616655 PMC2838936

[41] Hollingworth R, Grand RJ. Modulation of DNA damage and repair pathways by human tumour viruses. Viruses. 2015;7(5):2542–91. 10.3390/v7052542 26008701 PMC4452920

